# Predominant heterotrophic diazotrophic bacteria are involved in *Sargassum* proliferation in the Great Atlantic *Sargassum* Belt

**DOI:** 10.1093/ismejo/wrad026

**Published:** 2024-01-10

**Authors:** Matéo Léger-Pigout, Elisabeth Navarro, Frédéric Ménard, Sandrine Ruitton, François Le Loc’h, Sophie Guasco, Jean-Marie Munaron, Delphine Thibault, Thomas Changeux, Solène Connan, Valérie Stiger-Pouvreau, Thierry Thibaut, Valérie Michotey

**Affiliations:** Aix Marseille Univ, Université de Toulon, CNRS, IRD, MIO, Marseille, France; Aix Marseille Univ, Université de Toulon, CNRS, IRD, MIO, Marseille, France; Aix Marseille Univ, Université de Toulon, CNRS, IRD, MIO, Marseille, France; Aix Marseille Univ, Université de Toulon, CNRS, IRD, MIO, Marseille, France; Univ Brest, CNRS, IRD, Ifremer, LEMAR, F-29280 Plouzane, France; Aix Marseille Univ, Université de Toulon, CNRS, IRD, MIO, Marseille, France; Univ Brest, CNRS, IRD, Ifremer, LEMAR, F-29280 Plouzane, France; Aix Marseille Univ, Université de Toulon, CNRS, IRD, MIO, Marseille, France; Aix Marseille Univ, Université de Toulon, CNRS, IRD, MIO, Marseille, France; Univ Brest, CNRS, IRD, Ifremer, LEMAR, F-29280 Plouzane, France; Univ Brest, CNRS, IRD, Ifremer, LEMAR, F-29280 Plouzane, France; Aix Marseille Univ, Université de Toulon, CNRS, IRD, MIO, Marseille, France; Aix Marseille Univ, Université de Toulon, CNRS, IRD, MIO, Marseille, France

**Keywords:** Sargassum, N2 fixation, nitrification, denitrification, GASB, isotopy, Vibrio spp

## Abstract

Since 2011, the Caribbean coasts have been subject to episodic influxes of floating *Sargassum* seaweed of unprecedented magnitude originating from a new area “the Great Atlantic *Sargassum* Belt” (GASB), leading in episodic influxes and mass strandings of floating *Sargassum*. For the biofilm of both holopelagic and benthic *Sargassum* as well as in the surrounding waters, we characterized the main functional groups involved in the microbial nitrogen cycle. The abundance of genes representing nitrogen fixation (*nifH*), nitrification (*amoA*), and denitrification (*nosZ*) showed the predominance of diazotrophs, particularly within the GASB and the Sargasso Sea. In both location, the biofilm associated with holopelagic *Sargassum* harboured a more abundant proportion of diazotrophs than the surrounding water. The mean δ^15^N value of the GASB seaweed was very negative (−2.04‰), and lower than previously reported, reinforcing the hypothesis that the source of nitrogen comes from the nitrogen-fixing activity of diazotrophs within this new area of proliferation. Analysis of the diversity of diazotrophic communities revealed for the first time the predominance of heterotrophic diazotrophic bacteria belonging to the phylum *Proteobacteria* in holopelagic *Sargassum* biofilms. The *nifH* sequences belonging to *Vibrio* genus (*Gammaproteobacteria*) and *Filomicrobium* sp. (*Alphaproteobacteria*) were the most abundant and reached, respectively, up to 46.0% and 33.2% of the community. We highlighted the atmospheric origin of the nitrogen used during the growth of holopelagic *Sargassum* within the GASB and a contribution of heterotrophic nitrogen-fixing bacteria to a part of the *Sargassum* proliferation.

## Introduction

Since 2011, blooms of the holopelagic brown macroalgae *Sargassum* spp. have been observed in the tropical North Atlantic Ocean, causing significant beach strandings along the Caribbean coasts. The presence of *Sargassum* is a permanent feature of the North Atlantic, where massive quantities are trapped in the Sargasso Sea by large-scale hydrodynamics. Holopelagic *Sargassum* has flourished naturally in this area despite the nutrient-poor and low-productivity characteristics of the surrounding waters [1]. Several factors could explain this apparent paradox: first, *Sargassum* growth is driven by new production in the neritic waters of the western North Atlantic Ocean and the Gulf of Mexico, where mutualistic relationships with fish could contribute to nutrient supply [2]. Second, seasonal transport of nutrient-rich and productive *Sargassum* from the Gulf of Mexico is facilitated by large-scale currents such as the Loop Current and the Gulf Stream, which enter the Sargasso Sea [3]. Third, nitrogen fixation by the *Cyanobacteria Dichothrix fucicola*, an epiphyte on *Sargassum*, has been shown to provide a significant portion (2%–32%) of the nitrogen demand for the holopelagic *Sargassum* in the Sargasso Sea [4, 5]. Recently N fixation rates associated with *Sargassum* ranging from 0 to 30 916 μmol.N.m^−2^.d^−1^ have been recorded in the North Atlantic [6]. This nitrogen fixation contributes to variable δ^15^N enrichment in *Sargassum* tissues, ranging from approximately 8‰ to −2‰ [7, 8].

*Sargassum* stranding occurs along the Gulf of Mexico coast, resulting in negative economic [9-13] and environmental [13-15] impacts. The development of *Sargassum* in this region has been attributed to increasing nitrogen inputs from various land-based sources, including the Mississippi River and its tributaries [3, 16]. The higher δ^15^N enrichment of *Sargassum* in the Gulf of Mexico (in the range of 5‰–8‰) compared to that observed in the Sargasso Sea may be related to the urbanization of the Texas coast and the Mississippi River plume [8].

In 2010–2011, a significant biomass of *Sargassum* was discovered in a new region of the tropical Atlantic Ocean south of the Sargasso Sea, where it had never previously occurred in such abundance [17, 18]. Since then, the seaweed has flourished, forming a new consolidated region called the “Great Atlantic *Sargassum* Belt” (GASB), which extends from the coasts of Africa to South and Central America. These drifting seaweeds are carried by ocean currents and winds, aggregate at the surface to form visible rafts that can be detected by satellite remote sensing across the transatlantic region. As a result, large quantities, reaching up to 20 million tons, regularly wash up on the coasts of the Caribbean islands, northern Brazil, Guyana, and West Africa [19]. This represents an emerging threat to areas already under anthropogenic pressure. As in the Gulf of Mexico, the rapid decomposition of stranded *Sargassum* in these regions poses significant health risks to local populations [20] and economy [21] due to the release of hydrogen sulphide (H_2_S), a toxic and corrosive gas [22].

These multiregional transboundary *Sargassum* influxes in the GASB do not appear to result from a single or simple cause, but rather from complex combination of causal factors. These include higher water temperatures [23, 24], nutrient inputs from rivers in the region, such as the Amazon [8], and upwelling of nutrient-rich deep water [19, 25]. Nevertheless, these hypotheses do not fully explain the extent of proliferation [23, 26-29], and significant gaps remain in our knowledge and understanding. For example, the potential role of *Sargassum* microbial biofilms in promoting algal growth has not been thoroughly investigated in the GASB.

In a 2020 study [30], an analysis of samples from the GASB and revealed the presence of four distinct *Sargassum*-associated microbial biofilms, with only one shared with the Gulf of Mexico counterpart [30, 31]. A subsequent study in 2023 [32] also showed biogeographic patterning of the holopelagic *Sargassum* microbiome and identified biomarker genera indicative of *Sargassum natans* I/VIII and *Sargassum fluitans* III. This suggests that the new location may select for different microbial members within the *Sargassum* biofilm. As nitrogen was identified as a potential limiting factor for *Sargassum* growth, we thus investigate the nitrogen cycle and the interactions between *Sargassum* and its prokaryotic biofilm partners in the GASB.

As a result, our study quantified the contribution of key microbial members of the nitrogen cycle, such as diazotrophs, nitrifiers, and denitrifiers, responsible for the entry, transformation, and exit of assimilable nitrogen, from holopelagic *Sargassum* and its surrounding waters. This large-scale study extended from the GASB to the Caribbean Islands and the southern Sargasso Sea, including samples of benthic *Sargassum* in the Caribbean. The study focused on the diversity of diazotrophs and analysed their relationships with the δ^15^N values of the seaweed and the particulate organic matter (POM), including phytoplankton, in the adjacent water.

This approach aimed to investigate the relative contributions of these microbial members to the growth of *Sargassum* in the GASB and Caribbean coastal zones and ultimately identified additional key microbial players that could contribute to the proliferation of *Sargassum*.

## Materials and methods

### Sampling campaigns

**Figure 1:**
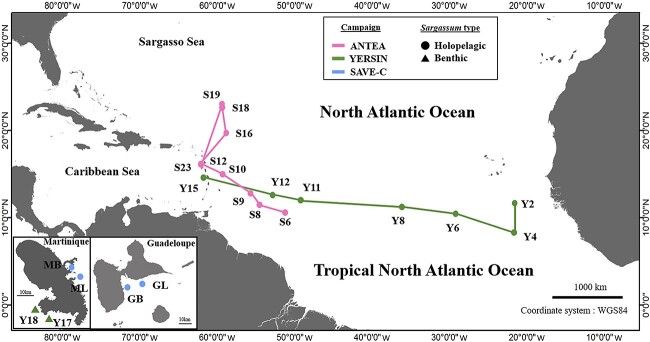
Location of sampling stations (*Sargassum* and surrounding waters). ANTEA (June 19 to July 13, 2017, S-stations), YERSIN (10–24 October 2017, Y-stations), SAVE-C (7–17 July 2021, M- or G-stations) campaigns. Holopelagic *Sargassum* samples are represented by dots and benthic samples by triangles.

Twenty sampling stations were set up during two sea cruises and one coastal mission in the French West Indies (Fig. 1). The Western Atlantic-*Sargassum* expedition (https://doi.org/10.17600/17004300) took place aboard the N/O ANTEA from 19 June to 13 July 2017 and explored the GASB as well as the Sargasso Sea (25°N). The Transatlantic-*Sargassum* expedition (https://doi.org/10.17600/17016900) took place aboard the M/V YERSIN from 6 to 24 October 2017 from the Cape Verde Islands, crossing the Atlantic between 8 and 12°N to the island of Martinique within the GASB. The SAVE-C coastal sampling period along the coasts of Martinique and Guadeloupe took place from 7 to 17 July 2021. Despite the fact that sampling missions were performed at different years and seasons (Antea and Yersin in 2017 and SAVE-C in 2021, and spring/summer for Antea and SAVE-C and Autumn for Yersin), water temperatures were around 28°C and showed less than 1°C variation (Antea 27.6°C to 28.7°C, Yersin 27.4°C to 27.7°C, and Save-C 27.42°C to 28.28°C). At each station, apical sections of stems with leaves and small bladders (pneumatocysts) of the three holopelagic morphotypes (*S. natans* I and VIII and *S. fluitans* III identified as described previously [33]) as well as water samples surrounding the rafts were collected in at least triplicate. The typology of the sampled *Sargassum* rafts [34] was also recorded.

To analyse the biofilm and associated microbial communities growing on *Sargassum* (*n* = 159), approximately 5 g wet weight of *Sargassum* was placed in a 100 -cm^3^ cup, filled with 70 cm^3^ of sterile seawater and sonicated for 30 s (46 kHz, 30 W) to dissociate the bacterial biofilm from its support [30]. The water containing the dissociated biofilm was then filtered through 0.22 -μm filters (nitrocellulose membrane, 47 mm), and the filters were stored at −20°C. For the microbial communities in the surrounding waters, 4 l of surface water was collected adjacent to the sampled *Sargassum* using a clean bucket. Planktonic microorganisms were recovered by filtering the water through a 0.22 -μm nitrocellulose membrane (47 mm, *n* = 83) in at least in triplicate for each station. The filters were stored at −20°C until further analysis.

Nitrogen stable isotope analysis (δ^15^N) was conducted on *Sargassum* thallus from the three morphotypes with at least three samples per morphotype. The samples (*n* = 262) were promptly stored in silica gel. At a particular location, stems, leaves, and bladders were examined separately. During the sampling period in Martinique and Guadeloupe, we tested the impact of biofilm microbial communities on the isotopic composition by analyzing samples with (*n* = 45) and without (*n* = 34) biofilm, which has been removed through sonication. The isotopic composition of the POM, which includes planktonic microorganisms, was acquired by filtering at least 20 l of the surrounding water in triplicate through preweighed Whatman GF/F glass microfiber 0.7-μm filters (precombusted at 500°C for 4 h, *n* = 110). The filters were subsequently dried and stored in silica gel until further analysis.

### Microbial analysis

To better characterize the nitrogen cycle in the *Sargassum* ecosystem of GASB compared to other areas, microbial analysis was performed on the frozen samples. The DNA collected on nitrocellulose membranes from microorganisms of the *Sargassum* biofilm as well as the water surrounding the *Sargassum* were extracted using a published protocol [30] for the Antea and Yersin cruises samples, and DNAeasy®PowerLyser®PowerSoil®Kit (Qiagen) was used for SAVE-C cruise samples. Diazotrophs, nitrifiers, and denitrifiers were then studied through the relative abundance of a selection of their marker genes (*nifH*, *amoA*, and *nosZ*, respectively) relatively to ribosomal gene (16S rRNA genes). qPCR were performed on DNA from all biofilms and surrounding water samples. Denitrifiers quantification (*nosZ* gene) was performed with the primers *nosZ* 2F [35]—*nosZ* 1897R [36]. Bacterial nitrifiers quantification (*amoA* gene) was performed with the primers *amoA*-1F—*amoA*-2R [37] and the primers arc-*amoA* F100 mod—arc-*amoA* R336 mod [38] were used to amplify the archeal *amoA* gene. The mixes (20 μl) used in these qPCRs were composed of EvaGreen 2X (10 μl Bio-Rad®), primer F/R (0.5 μl), DNA (2 μl), and H_2_O (7 μl). We used the following program during qPCR of the genes *nosZ*, bacterial *amoA*, and archeal *amoA*: an initial denaturation (98°C for 5 min, 2 min, 2 min, respectively) followed by 40 cycles each comprising a denaturation step (98°C for 15 s–5 s–5 s, respectively), hybridization (64°C–58°C–62°C for 15 s, respectively), elongation (72°C for 40 s–1 min–1 min, respectively), and a final elongation (95°C for 10 s). For qPCR of the *nifH* gene, the primers used commonly for the nested PCRs used in the *nifH* gene amplicon sequencing analysis [39] were not compatible with this analysis. Instead, we used the PolF/PolR primers [40], frequently used in marine and terrestrial environments. Validation of PolF/PolR primers coverage against GenBank nr database was performed using all combinations offered by the degenerate sequences of these two primers. A coverage of all the diazotroph taxa was observed. Moreover, these primers covered more than 90% of the relative abundance obtained after *nifH* gene amplicon sequencing analysis (Supplementary Table 1). We also tested their amplification efficiency on proteobacterial and cyanobacterial fragments obtained in our study and that had been previously inserted into plasmids. In these tests, the calibration curve exhibited good characteristics (*R*^2^ = 0.99; *E* = 91.8%) and was used as the standard range for the entire analysis (Supplementary Fig. 1). The mixes (20 μl) used in the *nifH*-qPCR were composed of EvaGreen 2X (10 μl, Bio-Rad®), PolF/PolR (0.5 μl), DNA (2 μl), and H_2_O (7 μl). We used the following program during qPCR: an initial denaturation (98°C for 2 min) followed by 45 cycles each comprising a denaturation step (98°C for 5 s), hybridization (55°C for 15 s), elongation (72°C for 15 s), and finally a melting curve (from 65°C to 95°C with an increment of 0.5°C every 5 s). Each qPCR was validated following the observation of a correct melting curve, considering the large variability in *nifH* gene size [41]. Quantification of the 16S rRNA genes was performed as described previously [30].

The diversity of diazotroph was studied at each station (biofilm and surrounding water *n* = 35) after pooling replicate DNA extracts. For *nifH* gene amplicon sequence analysis, MiSeq (Illumina) 2 × 250 bp sequencing of the *nifH* gene was performed (Genewiz®) following nested PCR amplification, with *nifH*1/*nifH*2 and *nifH*3/*nifH*4 sets [39] and sequencing. Primer deletion, quality filtering, dereplication, error correction, sample inference, merging of paired reads, chimaera removal, and taxonomic classification of raw sequences were performed using R software (4.1.2) and dada2 (1.22.0) pipeline [42]. Approximately 5 000 000 reads were obtained from the 35 samples (145 000 reads per sample on average). A first taxonomic assignment was performed against a *nifH* database (June 2017 version) [43] in dada2 format (https://doi.org/10.5281/zenodo.3958370). As many amplicon sequence variants (ASVs) were not assigned or did not have a precise taxonomic assignment for this study, we enriched the database with assigned sequences specific to our dataset. Each ASV that did not have a taxonomic assignment at the class level was subjected to BLAST analysis against the GenBank nr database. Sequences that matched with ASVs at a high percentage identity (>85%) were integrated into the initial database. A second taxonomic analysis was performed with this in-house database. In total, 942 ASVs were identified via this analysis (Supplementary Data 1). However, this method was not able to assign all the ASVs to the class level. We therefore performed the class phylogeny of all unassigned ASVs representing at least 1% of each sample using Clustal Omega alignment (https://www.ebi.ac.uk/Tools/msa/clustalo/) and NJ tree constructed using SeaWiew software (https://doua.prabi.fr/software/seaview) (Supplementary Fig. 2). Raw sequence data are available at the NCBI Sequence Read Archive under bio project accession #PRJNA1017983.

### Determination of δ^15^N in *Sargassum* and POM samples

For pieces of *Sargassum*, approximately 2 g of sample dried powder was weighed in tin capsules for isotopic analysis. For POM, samples were duplicated: Only the subsamples that were not treated were analysed for δ^15^N. Stable isotope analyses were performed by continuous flow on a Flash EA 2000 elemental analyser (Thermo Scientific, Milan, Italy) coupled to a Delta V Plus, isotope ratio mass spectrometer (Thermo Scientific, Bremen, Germany) at the Pôle Spectrométrie Océan (IUEM-UBO, Plouzané, France). The reference materials (USGS-61, USGS-62, and USGS-63) and an in house standard Thermo-Acetanilide were used for isotopic corrections and to assign the data to the appropriate isotopic scale: USGS-61 (certified values: δ^15^N = −2.87‰ ± 0.04‰ and measured values: δ^15^N = −2.89‰ ± 0.06‰), USGS-62 (certified values: δ^15^N = +20.17‰ ± 0.06‰ and measured values: δ^15^N = +20.17‰ ± 0.08‰), and USGS-63 (certified values: δ^15^N = +37.83‰ ± 0.06‰ and measured values: δ^15^N = +37.84‰ ± 0.10‰). Results were reported in the δ unit notation and expressed as parts per thousand relative to the international standards atmospheric N_2_ for nitrogen. Analytical precision based on replicate measurements (after every five samples) of Thermo-Acetanilide was <0.1 for δ^15^N values.

### Statistics

All statistics were performed using R software (4.1.3). To define how the abundances of the nitrogen cycle members and the δ^15^N varied between the different microbiomes, the normality of the groups was first tested (*stats* package, 4.1.2) using Shapiro–Wilk tests. Then nonparametric Kruskal–Wallis analysis followed by Nemenyi tests (*PMCMRplus* package, 1.9.6), Welch’s *t*-tests, and Wilcoxon tests were performed. Plots and associated 95% confidence intervals of the mean (2000 bootstraps) were drawn using the *ggplot2* package (3.4.2). To assess the variation of diazotrophic communities according to microbiomes, we first replaced the zeros in the ASV table using the *zCompositions* package. The ASV table was then transformed into a compositional log-ratio (clr) matrix to mitigate the compositional nature of the data. An Aitchison distance matrix was then computed using the *phyloseq* package (1.38.0). The NMDS analysis was performed on the Aitchison distance matrix using the *vegan* package (2.6.4), employing a configuration with two dimensions (*k* = 2) and a maximum of 500 iterations (trymax = 500), with an initial configuration generated after 200 tries (try = 200). In addition to NMDS, a Hierarchical Ascendant Classification (HAC) based on the Aitchison distance matrix was performed (method = complete) using the *stats* package (4.1.2). The dendrograms obtained were cut into four clusters using the cutree function in the *stats* package (4.1.2). Dunn’s index was calculated using the package *fpc* (2.2.10).

## Results

### Relative abundance of bacteria involved in nitrogen cycle processes

**Figure 2:**
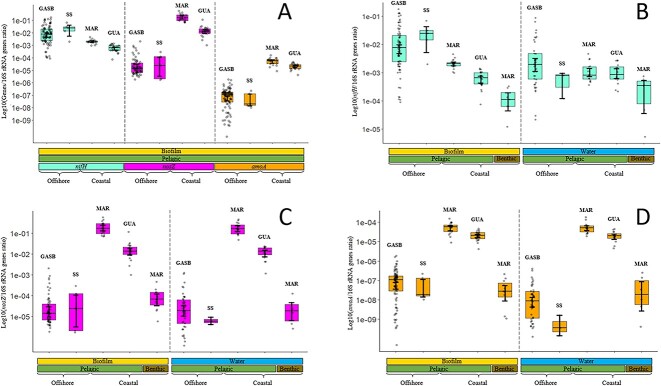
Log_10_ evolution of the ratio of *nifH*, *nosZ*, and *amoA* gene abundances to 16S rRNA genes abundance in different microbiomes. For each graph, samples are categorized according to their origins: biofilm or water, pelagic or benthic, open ocean or coastal, samples. Each grey point corresponds to one data item. The 95% confidence intervals of the mean are shown. (A) Relative abundance of the *nifH*, *nosZ*, and *amoA* genes in the *Sargassum* biofilm. (B) Relative abundance of the *nifH* gene across microbiomes. (C) Relative abundance of the *nos*Z gene across microbiomes. (D) Relative abundance of the *amoA* gene across microbiomes. SS, Sargasso Sea; MAR, Martinique; GUA, Guadeloupe.

In the holopelagic *Sargassum* biofilm from the offshore samples (GASB and Sargasso Sea), diazotrophs (*nifH/*16S rRNA genes ratio) were found to comprise 2% ± 1% and were significantly dominant over denitrifiers and nitrifiers (approximately 10^3^ to 10^5^ times higher, *P <* .001) (Fig. 2A). However, biofilm composition underwent significant changes in coastal samples, and varying trends were observed among the microbial communities. Diazotrophs exhibited a significant 10-fold decrease (*P <* .05) in abundance between offshore and coastal samples, reaching approximatively 0.1%. In contrast, nitrifiers and denitrifiers showed a substantial increase, respectively, 10^2^- and 10^3^-fold higher (*P <* .001). Consequently, in the holopelagic *Sargassum* biofilm collected from the coastal region of Martinique and Guadeloupe, denitrifiers became prevalent over diazotrophs (*P <* .001) and reached 9.7% ± 4.1% (*nosZ*/16S rRNA genes ratio). Within samples from the coastal area, the relative abundance of diazotrophs, nitrifiers, and denitrifiers in the holopelagic *Sargassum* biofilm was higher compared to that in the biofilm of benthic *Sargassum* collected on the same island (Fig. 2B–D). To investigate possible connections between holopelagic *Sargassum* and its biofilm, a comparison between relative abundance of the analysed genes collected from *Sargassum* biofilm and their surrounding water was performed. In offshore samples, relative abundance of diazotrophs or nitrifiers community between *Sargassum* biofilm and surrounding water showed significant differences (*P <* .05; Fig. 2B–D) in contrast to denitrifiers (Fig. 2C). In coastal areas, no difference was observed between the relative abundance of the analysed genes retrieved from holopelagic *Sargassum* or its surrounding waters. Consequently, conditions encountered within biofilms of holopelagic *Sargassum* in offshore samples (GASB and Sargasso Sea) seem to favour diazotroph and nitrifier development compared to the surrounding waters. This positive effect seems to disappear in coastal areas. To determine whether raft size, *Sargassum* morphotype, or geographic location affect biofilm composition, the relative abundances of the selected genes were studied among our extensive set of biofilm samples (Supplementary Data 2). Our results indicate that neither the morphotypes of holopelagic *Sargassum* nor raft types had a significant impact on the relative abundance of *nifH*, *nosZ*, and *amoA* genes in the biofilm within the GASB (Supplementary Fig. 3, *P >* .05). Gene abundance was found to be similar (*P >* .05; Fig. 2) in offshore samples.

### Determination of the source of nitrogen in *Sargassum* and POM of their surrounding waters

**Figure 3:**
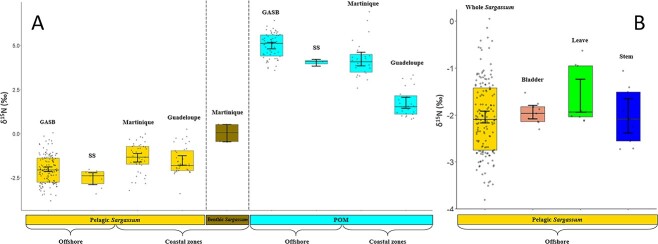
Evolution of δ^15^N (‰) as a function of the different sampled microbiomes. (A) Evolution of δ^15^N (‰) as a function of different sample types: holopelagic and benthic *Sargassum*, and POM and between open ocean and coastal areas samples. (B) Evolution of δ^15^N (‰) as a function of the different parts of the holopelagic *Sargassum* (all parts combined, bladders, leave, stem). GASB corresponds to the Great Atlantic *Sargassum* Belt. SS corresponds to Sargasso Sea. Each point corresponds to one data. The 95% confidence intervals of the mean are shown.

Isotopic analysis of δ ^15^N was conducted to examine the contribution of nitrogen by diazotrophs (Supplementary Data 3). Measurements of δ^15^N were performed on *Sargassum* and POM (including phytoplankton) from the same sample set (holopelagic *Sargassum* from offshore and coastal area, benthic *Sargassum*, *n* = 262). Analyses were performed on *Sargassum* with their entire biofilm or mostly removed by sonication, and on different parts of the seaweed at sampling stations located in the centre of the GASB (leave, stem, and bladder). The δ^15^N value of offshore holopelagic *Sargassum* (with biofilm) was extremely low (−2.04‰ on average) with no differences observed between GASB and Sargasso Sea samples (Fig. 3A). In the coastal zone, δ^15^N of holopelagic *Sargassum* (with biofilm) increased to reach about −1‰. Sonication of holopelagic *Sargassum*, to disassociate the biofilm, did not have any significant effect on δ^15^N of the seaweed (*P >* .05). Furthermore, in the centre of the GASB (Y11 station), δ^15^N of the stem, leaves, and bladders of the holopelagic *Sargassum* presented neither significant difference between them nor with the entire seaweed (*P >* .05) reflecting a common nitrogen source (Fig. 3B). Overall δ^15^N of holopelagic *Sargassum* (−2.04‰ on average) are significantly lower than those measured for the corresponding POM (5.06‰ on average) or that of benthic *Sargassum* (δ^15^N of about 0‰) (Fig. 3A). These results suggest that fixed N_2_ by microbial biofilm accounts for an important proportion of the assimilated nitrogen of the holopelagic *Sargassum* of GASB, with a constant source along the growth of the different parts of the thallus. However, the amount of fixed nitrogen decreased upon reaching the coastal Caribbean region.

### Biofilm containing an original diazotrophic community structure

**Figure 4:**
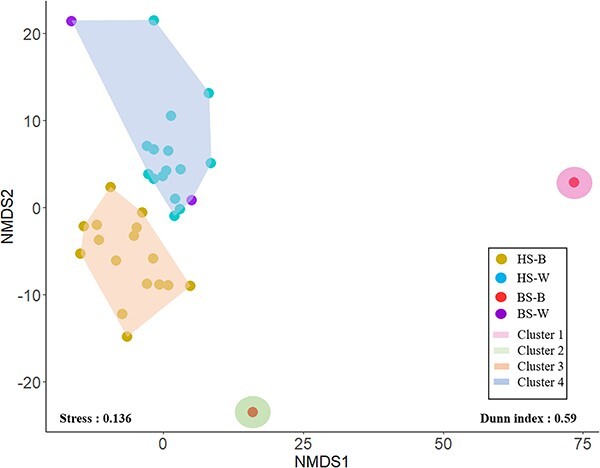
NMDS and HAC analysis of relative abundance of diazotrophs per station according to microbiomes. Each point represents a station. The groups correspond to holopelagic *Sargassum* biofilm (HS-B) and associated water (HS-W), benthic *Sargassum* biofilm (BS-B) and associated water (BS-W). The NMDS analysis (*S* = 0.125) was performed using an Aitchison distance matrix. The four clusters obtained with a HAC (Dunn index = 0.59; Supplementary Fig. 5) are represented by the coloured envelopes.

**Figure 5:**
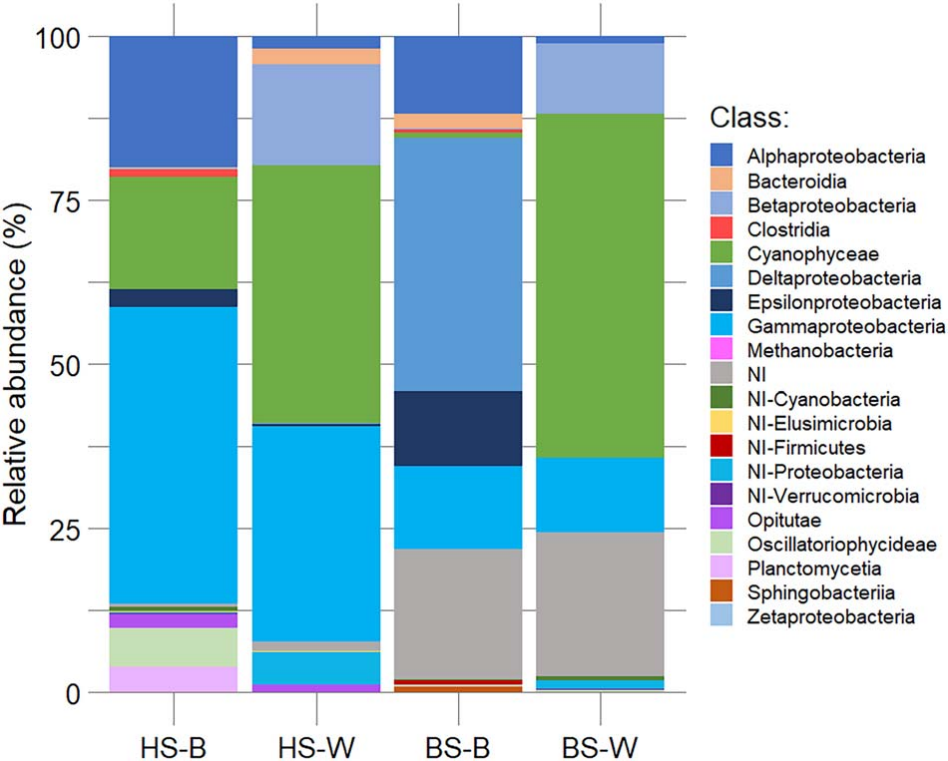
Relative abundances (%) of diazotroph classes in relation to microbiomes. The microbiomes shown correspond to the holopelagic *Sargassum* biofilm (HS-B) and associated water (HS-W), and the benthic *Sargassum* biofilm (BS-B) and associated water (BS-W). NI, not identified.

Analyses of *nifH* gene amplicon sequencing revealed differences in diazotroph compositions between holopelagic and benthic *Sargassum* biofilm (BS-B), or corresponding surrounding waters (Figs 4 and 5; Supplementary Fig. 3). In the offshore, holopelagic *Sargassum* biofilm communities were predominantly composed of noncyanobacterial diazotrophs (NCDs, 76.1% ± 13.2%), with *Cyanobacteria* representing only 23.9% ± 13.2% of the diazotroph community. NCDs are dominated by *Proteobacteria* that reached on average 68.3% ± 19.3% of the diazotroph community (Fig. 5; Supplementary Fig. 3). Additionally, there is a small percentage of *Planctomycetes* (4%) and *Firmicutes* phylum (1%). Among the NCDs, the two most abundant taxa (ASV) corresponded to a *Gammaproteobacteria* for which the closest sequence corresponds to *Vibrio* sp. (abundance among *nifH* up to 46.0%) and an *Alphaproteobacteria* that cluster with *Filomicrobium* sp. (abundance up to 33.2%). Among the cyanobacterial diazotroph of the holopelagic *Sargassum* biofilm, the two most taxa were affiliated to the genera *Mastigocoleus* (1.2% ± 0.9% in average, order *Nostocales*) and *Hyella* (1.3% ± 1.4% in average, order *Pleurocapsales*). The communities within the surrounding waters did not show such a marked difference between NCDs (essentially *Proteobacteria*) and cyanobacterial diazotrophs, they represented, respectively, 55.6% ± 16.6% and 39.4% ± 16.1%. The two most abundant NCD’s taxa belonged, for the first one to a nonidentified *Proteobacteria* (10.5% ± 15.4%) and for the second one to an *Alcaligenes* sp. (8.5% ± 7.2%). The two most abundant *Cyanobacteria* taxa in water were both affiliated to *Trichodesmium* sp. (27.3% ± 15.6% and 9.6% ± 11.4%, respectively). *Dichothrix fucicola* populations appeared as minor member of the diazotroph community, and their contribution ranged between 1% and 6.5% (S8 and S10 stations, Supplementary Fig. 4) in the GASB. The biofilm of benthic *Sargassum* showed a diazotroph community mainly dominated by *Proteobacteria*, representing up to 75% in the biofilm of *Sargassum* (Supplementary Fig. 3).

## Discussion

Because *Sargassum* could be found in oligotrophic environments with low nitrogen concentrations [8], it is crucial to determine the various nitrogen sources utilized by these macroalgae and the underlying mechanisms. Our study reveals the significance of N_2_ fixation compared to nitrification and denitrification in GASB, and this pattern changes when the holopelagic *Sargassum* arrives in neritic waters.

Previous research has identified N_2_ fixation activity associated with *Sargassum* from the Sargasso Sea and the Gulf of Mexico, by acetylene reduction assays [4-6, 44, 45] and N_2_ assimilation by δ^15^N analysis [8, 46, 47]. Our study demonstrates that in the entire GASB and in the southern region of the Sargasso Sea, holopelagic *Sargassum* spp. exhibit negative δ^15^N values that are significantly lower than those measured for POM collected in their surrounding water. Our findings suggest a nitrogen source from fixed nitrogen by biofilm and exclude the involvement of free-living diazotrophs. Additionally, our study reveals the N source coming from diazotrophic nitrogen fixation activity of the biofilm in the new proliferation area. However, the δ^15^N values are lower than those measured in previous studies conducted in the Sargasso Sea and the Gulf of Mexico [8, 46, 47] indicating a higher level nitrogen fixation activity and an increased involvement of diazotrophs in suppling nitrogen to *Sargassum* in GASB. Similar isotopic values (δ^15^N) reported throughout the different parts of the *Sargassum* indicate a uniform nitrogen source during seaweed growth along its transatlantic journey. Nitrogen transfer efficiency between biofilm and *Sargassum* was demonstrated by the identical δ^15^N values of *Sargassum* both with and without its biofilm.

Despite their ecological importance, the diversity of the diazotroph community associated with *Sargassum* has been poorly characterized until now. In microscopic studies from the 1980s, *Cyanobacteria* including *Dichothrix fucicola* and others belonging to the genus *Calothrix* were identified [4]. Since then, *Cyanobacteria* have been considered the main diazotrophs and the primary contributors to nitrogen availability for *Sargassum* [4, 5]. Our findings from the GASB demonstrate that this idea is overly restrictive. Heterotrophic *Proteobacteria*, not *Cyanobacteria*, were the predominant diazotrophs in *Sargassum* biofilms in the GASB. The previous phylogenetic studies of the holopelagic *Sargassum* biofilm (based on 16S rRNA genes), collected in the same area [30-32], and from beach stranding [48, 49] have also observed a low occurrence of *Cyanobacteria*. Additionally, this study demonstrates, for the first time, the prevalence of heterotrophic bacteria (NCD) in the diazotroph community of *Sargassum* biofilm. Diazotrophs played a major role in the nitrogen cycle of *Sargassum*, particularly in GASB and the Sargasso Sea, which are known for stronger oligotrophic conditions as compared to coastal areas, as the isotopic results have demonstrated. In the latter regions, diazotrophy reduced, and the abundance of denitrifiers increase, resulting in an agreement with previous findings regarding the increase in δ^15^N [50].

Our results also demonstrate that the diversity of diazotrophic bacterial communities in offshore is distinct between *Sargassum* biofilms and surrounding waters, as previously reported [30, 31] based on 16S rRNA genes bacterial communities. The greater abundance of diazotrophs and higher prevalence of NCD in the holopelagic *Sargassum* biofilm compared to the surrounding waters in offshore suggest potential reciprocal benefits. Independent of the level of bacterial community analysis, in the open ocean, *Sargassum* biofilms are colonized by specific populations reinforcing the potential occurrence of close metabolite exchange links between seaweeds and their biofilms.

The source of carbon required for the metabolism of heterotrophic nitrogen fixing bacteria identified here remains unknown. The photo(hetero)trophs and organic matter degraders mainly composed the microbial community of holopelagic *Sargassum* [32] and could be one of carbon source. Furthermore, several studies have revealed that *Sargassum* spp. is a significant source of dissolved organic carbon [51, 52], could be directly metabolized by heterotrophic diazotrophs within their biofilm, and should be an example of mutualism relationships. Within the NCDs, an ASV clustering with *Vibrio* spp. *nifH* sequences was found to be among the most abundant, suggesting a biogeochemical role for this taxon in addition to a potential health risks in the area [30, 32].

Bacterial nitrogen fixation is the main mechanism that supplies nitrogen to holopelagic *Sargassum* in the GASB. The environmental characteristics of the open ocean promote rich diazotrophic biofilm, consequently reducing the potential nitrogen limitation factor through bacterial/seaweed interactions. Further investigations should focus on other nutrients, such as phosphorus, which may also play an important role in *Sargassum* proliferation [53, 54].

Our study identifies the crucial role of heterotrophic diazotrophs in the nitrogen cycle in the GASB. Our results suggest that this community contributes reactive nitrogen at a significant and basin-wide scale due to the area’s vastness. Further research is necessary to investigate the complex interactions among diazotroph community, *Sargassum*, and the surrounding environment in order to fully understand nitrogen dynamics in this unique marine ecosystem.

## Supplementary Material

Supplementary_Figures_final_revisions_27-11-2023_wrad026

Leger-Pigout_et_al_2023_ISMEJ_Supplementary_Table_wrad026

Supplementary_data_1_wrad026

Supplementary_data_2_wrad026

Supplementary_data_3_wrad026

## Data Availability

The nucleotide sequence data generated and analysed during the current study are available from the NCBI BioProject database under the BioProject ID #PRJNA1017983. The data sets generated or analysed during this study are available in Supplementary Data 1–3.
