# Supplementary material for: Predominant heterotrophic diazotrophic bacteria are involved in Sargassum proliferation in the Great Atlantic Sargassum Belt
Source: ISME J. 2024 Jan 10;18(1):wrad026. doi: 10.1093/ismejo/wrad026 (PMC10833076; doi:10.1093/ismejo/wrad026)
Supplement: Supplementary_Figures_final_revisions_27-11-2023_wrad026 [file supplementary_figures_final_revisions_27-11-2023_wrad026.pdf]

Supplementary Figures:

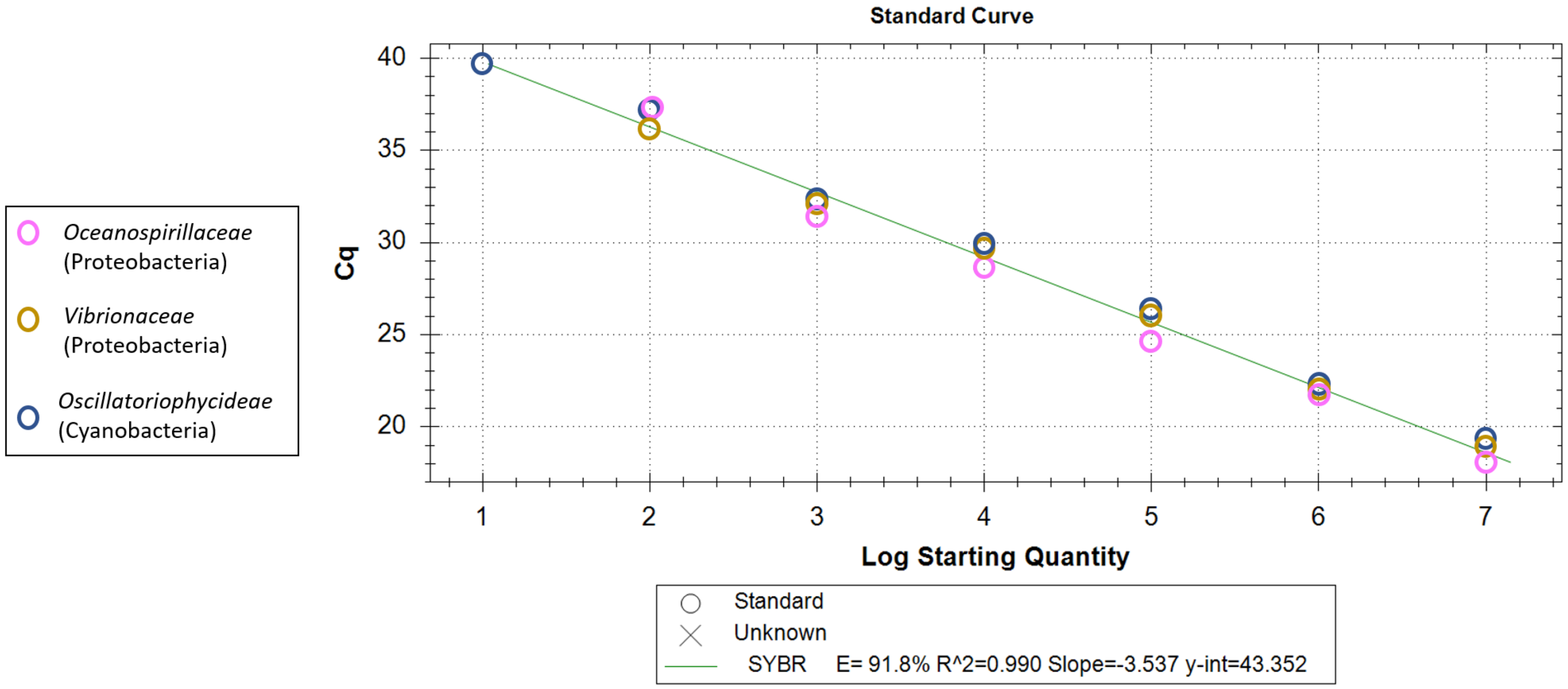

Supp. Fig. 1. Amplification of *nif*H clones. The standard curve is constructed by plotting the logarithm of the starting amount of template (DNA) against the C-value, obtained during the amplification of each dilution. The pink and brown circles correspond to Proteobacteria

(*Oceanospirillaceae* and *Vibrionaceae* respectively) and the green circles to Cyanobacteria (*Oscillatoriophycideae*). The efficiency value ($E = 10^{-1/slope}$), the coefficient of determination (R^2), the slope and the point of intersection on the y-axis, obtained when all 3 clones are used to make the calibration line, are given.

Supp. Fig. 2. NJ phylogenetic tree of unaffiliated ASVs following metabarcoding analysis. Proteobacteria (yellow background), cyanobacteria (green background), Bacteroidetes (blue background), Planctomycetia (purple background), Verrucomicrobia (pink background). The red lines show sequences belonging to the Alphaproteobacteria, the orange ones to the Gammaproteobacteria, the pink ones to the Deltaproteobacteria, the green ones to the cyanobacteria, the blue ones to the Bacteroidetes, the purple ones to the Planctomycetes and the brown ones to the Verrucomicrobia. The black circles indicate bootstrap values greater than or equal to 70. The identity of the ASVs is indicated in red.

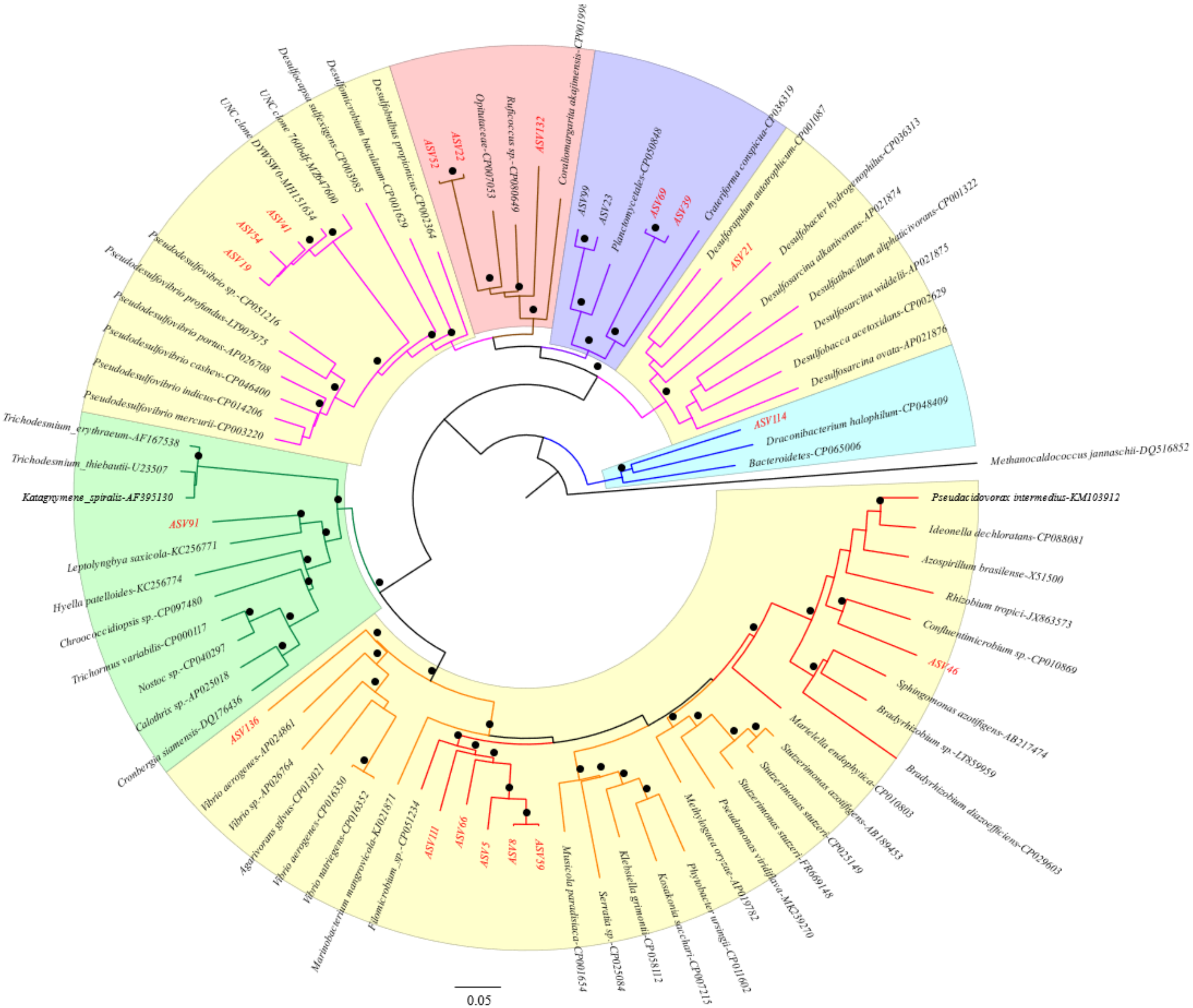

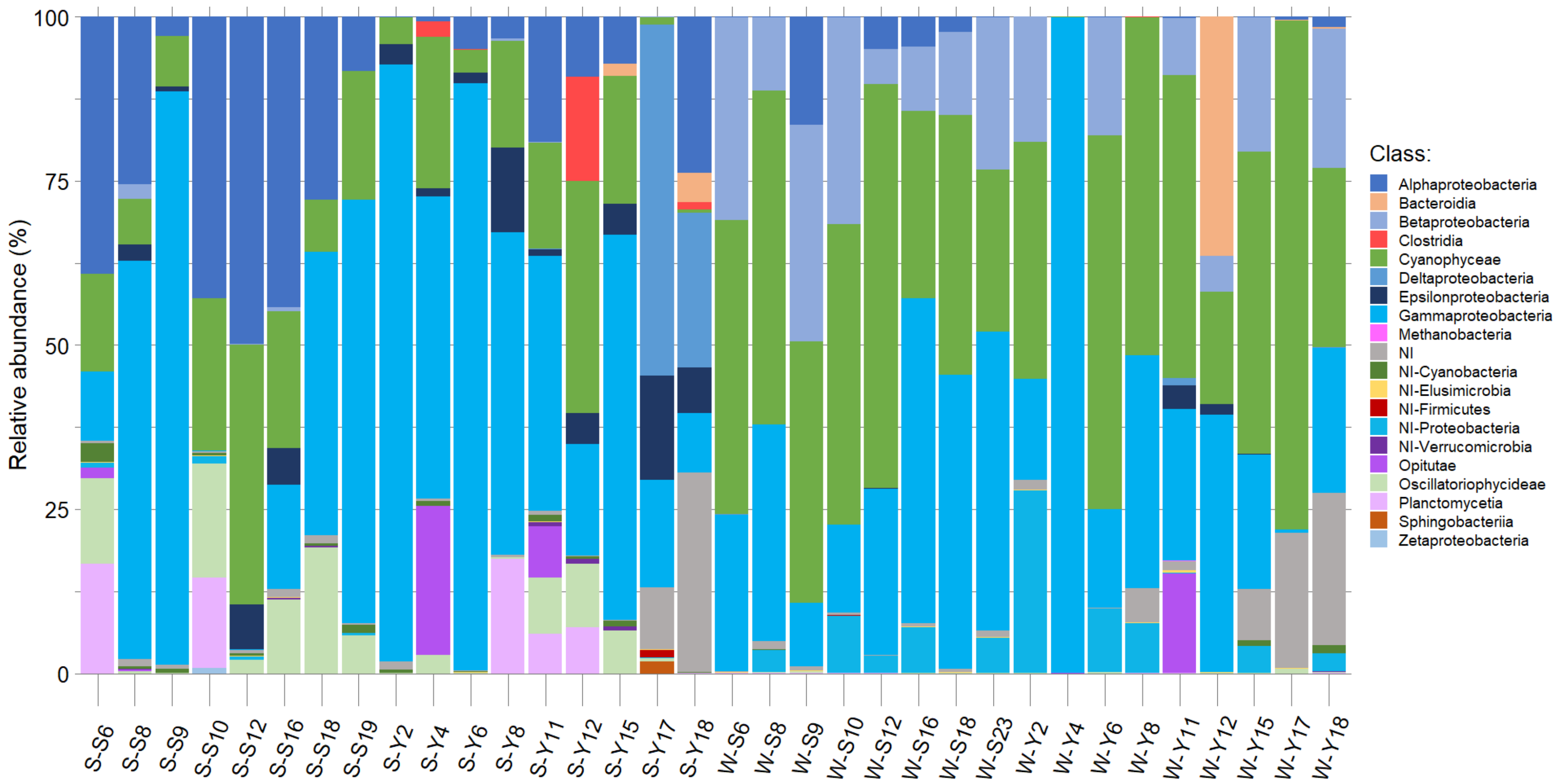

Supp. Fig. 3. Relative abundance (%) of diazotroph classes in relation to samples. For each sample, the first letter corresponds to the type of sample (S: *Sargassum* biofilm, W: water), the second letter corresponds to the campaign (A: Antea, Y: Yersin), and the number corresponds to the station sampled (the location of each station is shown in Fig. 1). Each color corresponds to a different class.

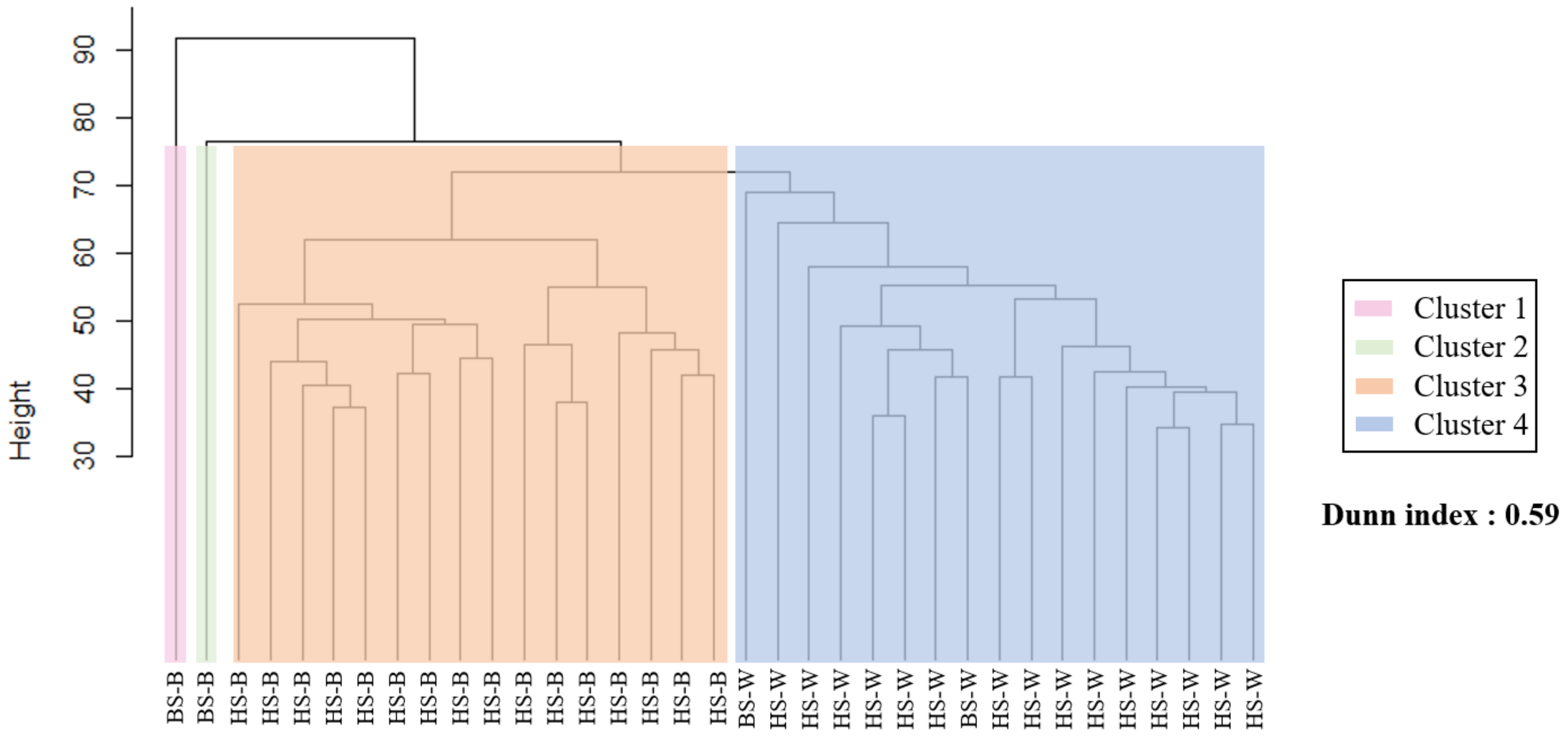

Supp. Fig. 4. Dendrogram obtained after hierarchical agglomerative clustering. Each branch corresponds to a sample. The sample type is indicated for each branch: holopelagic *Sargassum* biofilm (**HS-B**), associated water (**HS-W**), benthic *Sargassum* biofilm (**BS-B**) and associated water (**BS-W**). The 4 main clusters are shown in color on the dendrogram, along with the Dunn index associated with the analysis (Dunn index: 0.59).

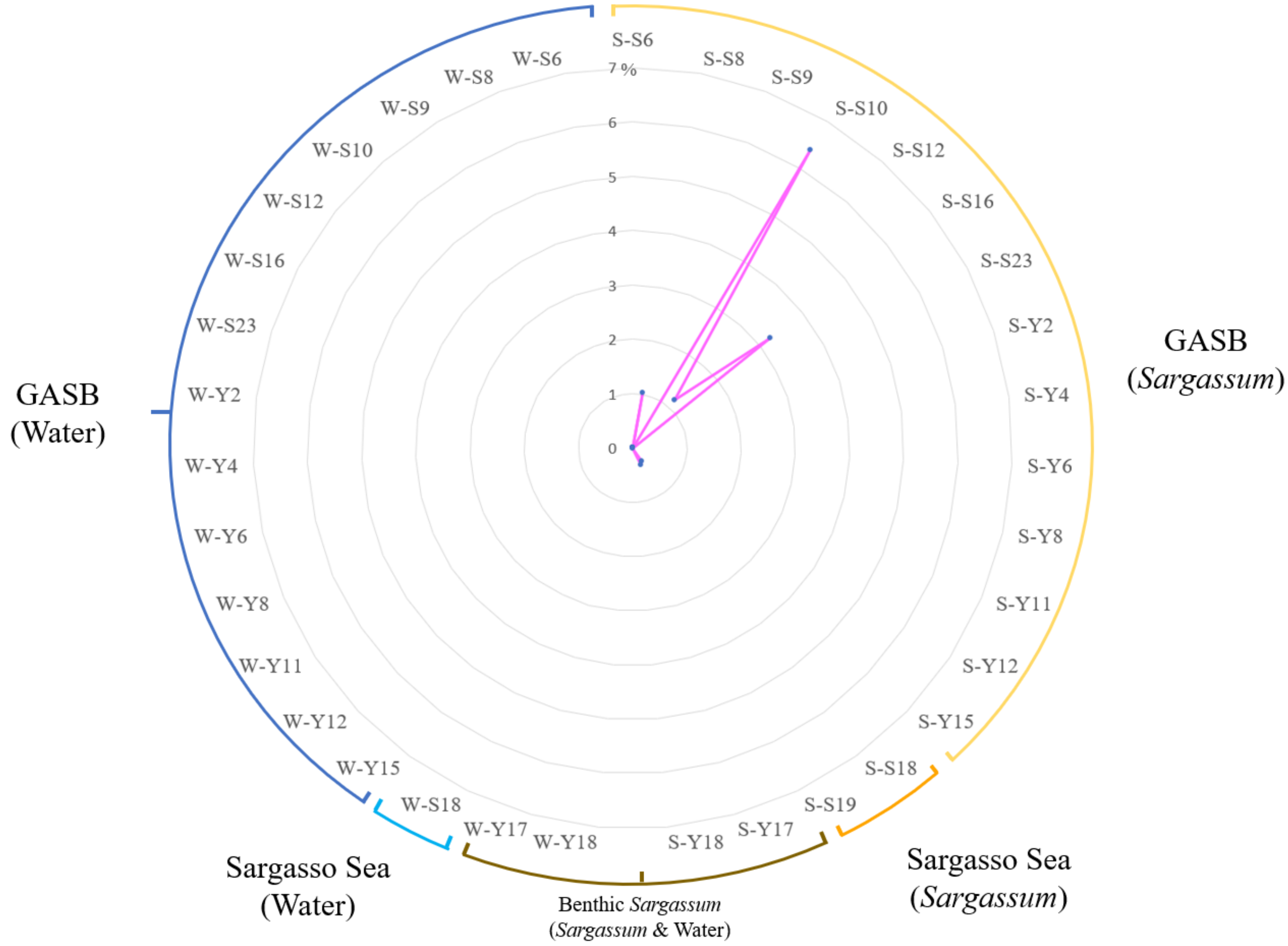

S-S6
S-S8
S-S9
S-S10
S-S12
S-S16
S-S23
S-Y2
S-Y4
S-Y6
S-Y8
S-Y11
S-Y12
S-Y15
S-S18
S-S19
S-Y17
S-Y18
W-Y18
W-Y17
W-S18
W-Y15
W-Y12
W-Y11
W-Y8
W-Y6
W-Y4
W-Y2
W-S23
W-S16
W-S12
W-S10
W-S9
W-S8
W-S6
7 %
6
5
4
3
2
1
0
GASB (Sargassum)
Sargasso Sea (Sargassum)
Benthic Sargassum (Sargassum & Water)
Sargasso Sea (Water)
GASB (Water)

Supp. Fig. 5. Evolution of the relative abundance of *Rivularia* according to the stations. Stations beginning with S- correspond to *Sargassum* biofilm samples and those beginning with W- to surrounding water samples. The location of the samples is also mentioned.
