# Supplementary material for: Predominant heterotrophic diazotrophic bacteria are involved in Sargassum proliferation in the Great Atlantic Sargassum Belt
Source: ISME J. 2024 Jan 10;18(1):wrad026. doi: 10.1093/ismejo/wrad026 (PMC10833076; doi:10.1093/ismejo/wrad026)
Supplement: Leger-Pigout_et_al_2023_ISMEJ_Supplementary_Table_wrad026 [file leger-pigout_et_al_2023_ismej_supplementary_table_wrad026.pdf]

Supplementary Table:

Kingdom	Phylum	Class	Validated Yes (Y) or No (N)	HS-B (% relative abundance)	HS-W (% relative abundance)	BS-B (% relative abundance)	BS-W (% relative abundance)
Bacteria	Bacteroidetes	Bacteroidia	N	0,1	2,5	2,8	0,1
Bacteria	Bacteroidetes	Sphingobacteriia	N	0,0	0,0	1,1	0,0
Bacteria	Cyanobacteria	Cyanophyceae	Y	17,1	39,8	1,1	67,0
Bacteria	Cyanobacteria	Oscillatoriophycideae	Y	6,1	0,1	0,3	0,6
Bacteria	Cyanobacteria	NI-Cyanobacteria	Y	0,8	0,1	0,1	0,8
Bacteria	Elusimicrobia	NI-Elusimicrobia	N	0,0	0,1	0,1	0,0
Bacteria	Firmicutes	Clostridia	N	1,2	0,0	0,7	0,0
Bacteria	Firmicutes	NI-Firmicutes	N	0,0	0,0	0,8	0,0
Bacteria	Planctomycetes	Planctomycetia	N	3,8	0,0	0,0	0,0
Bacteria	Proteobacteria	Alphaproteobacteria	Y	20,1	2,0	14,9	1,4
Bacteria	Proteobacteria	Betaproteobacteria	Y	0,2	15,5	0,0	13,6
Bacteria	Proteobacteria	Deltaproteobacteria	Y	0,0	0,1	48,0	0,1
Bacteria	Proteobacteria	Epsilonproteobacteria	N	2,8	0,4	14,2	0,0
Bacteria	Proteobacteria	Gammaproteobacteria	Y	45,2	33,4	15,9	14,5
Bacteria	Proteobacteria	Zetaproteobacteria	N	0,0	0,0	0,0	0,0
Bacteria	Proteobacteria	NI-Proteobacteria	Y	0,2	5,2	0,1	1,8
Bacteria	Verrucomicrobia	Opitutae	Y	2,0	1,0	0,0	0,0
Bacteria	Verrucomicrobia	NI-Verrucomicrobia	Y	0,1	0,0	0,0	0,0
Archaea	Euryarchaeota	Methanobacteria	N	0,0	0,0	0,0	0,0
			**Primer coverage (%)**	**92,0**	**97,1**	**80,3**	**99,8**

Supp. Table. 1. Coverage of PolF/PolR primers on the data of this study. Class validation is denoted by Y if the class is covered by the primers and N if not. The abundances of the different classes (considering only the identified sequences) and the coverage of the primers are also noted according to the different microbiomes.
